# The experience of pregnancy in the COVID-19 pandemic

**DOI:** 10.61622/rbgo/2025rbgo8

**Published:** 2025-03-17

**Authors:** Mariana Corniani Lopes, Cheryl Tatano Beck, Zelina Hilária de Souza Rosa, Erika de Sá Vieira Abuchaim

**Affiliations:** 1 Universidade Federal de São Paulo Escola Paulista de Enfermagem São Paulo SP Brazil Escola Paulista de Enfermagem, Universidade Federal de São Paulo, São Paulo, SP, Brazil.; 2 University of Connecticut School of Nursing Connecticut United States University of Connecticut, School of Nursing, Storrs, Connecticut, United States.

**Keywords:** Pregnancy, Pregnant women, COVID-19, Mental health, Pandemics

## Abstract

**Objective:**

To describe women's experience of pregnancy during the COVID-19 pandemic.

**Methods:**

A qualitative study conducted in a private maternity hospital, from May, 2020 to November, 2021, with women aged ≥ 18 years, gestational age ≥ 36 weeks at birth and ≥ 24 hours post-partum. Data collected through semi-structured interviews, recorded, transcribed, and analyzed adopting Krippendorff's Content Analysis as theoretical-methodological framework.

**Results:**

Four main themes emerged: Fear, Taking care and celebrating pregnancy: adjusting to the new reality, Harms of Isolation, and Benefits of Isolation. The fear of contamination and its impact on the health of mother and child resulted in the adoption of severe social isolation, including from those considered sources of support by the expecting mother. Overwhelmed, some of the participants reported loneliness and psychic suffering. The opportunity to focus on the pregnancy, the preparations for the arrival of the child, and the family made isolation a beneficial and positive period for other women.

**Conclusion:**

The experience of pregnancy in the Pandemic was an event outside of the ordinary and common. The expecting mother faced her worst fears on a daily basis and attended prenatal care, in order to ensure her child would be born healthy. The celebration of the baby's life, amid so many deaths, had to be adjusted to the virtual environment. It was a tense, solitary, and ambiguous period, which demanded a lot from the mental health of some participants, but to others, brought advantages that would not have been possible in different times.

## Introduction

Initially, the obstetric population was not considered a risk group for the development of serious COVID-19 symptoms. However, with the appearance of new evidence, this opinion was revised and pregnant women were classified as a significant risk population. In April, 2020, pregnant and postpartum women were officially included in the COVID-19 risk group.^(1-3)^

Brazil was one of the countries where the most pregnant women died due to lack of assistance. In the beginning of the pandemic, one in every five pregnant and postpartum women who died from COVID-19 did not have access to intensive care units (ICU) and 32.3% were not intubated.^(4-8)^

These high rates were associated with chronic and structural healthcare issues in Brazil, such as poor prenatal care and lacking resources and qualified healthcare professionals. These conditions were aggravated during the pandemic, with access to intensive hospital care (ICU) and proper ventilatory support being the main obstacles contributing to the increased mortality of mothers in the country.^(9,10)^

The COVID-19 pandemic was a historical event whose long-term impact on people's mental and physical health, particularly on expecting mothers who had to deal with increased work, family, and household demands without the necessary familial and social support, is not yet completely clear.^(4,7)^

The pregnancy-puerperal cycle is a phase that encompasses important events in the life of a woman and her family. The general and specific changes in the woman's body, combined with social and cultural changes, become a major challenge and require adjustments in various spheres of her life. It was believed that pregnancy was a period of fulfillment and joy for women; however, studies conducted in the last decades reveal that this can be a dark time for some of them, increasing the risk of developing mental disorders with severe consequences for maternal health, especially in the context of motherhood.^(4)^

The rates of mental disorders in perinatal mothers were already considered a public health issue, a concern in the agenda of international health agencies even before the COVID-19 pandemic, demanding promotion, prevention, and support actions for maternal mental health.^(4)^

In this setting, this study aimed at discovering, in the view of the women themselves, how the experience of being pregnant in the context of a global pandemic caused by an unknown virus was.

## Methods

This is a qualitative study conducted in two maternity hospitals in a supplementary health network that cares for normal-risk and high-risk pregnant women, located in the city of São Paulo. Combined, these maternity hospitals count with 14 beds for obstetric pathology, 103 postpartum beds, 93 neonatal intensive care unit (ICU) beds, and performed an average of 730 births/month at the time of the study. The study population consisted of women fluent in Brazilian Portuguese, aged ≥18 years, hospitalized in a rooming-in system, ≥24 hours postpartum, regardless of the type of birth. Postpartum women who tested positive for Covid-19, hospitalized for miscarriage, fetal death, malformed fetuses, stillbirth, or on palliative care were not included. The sample was defined by temporal convenience from November, 2020 to May, 2021. This period was selected due to the worsening of the pandemic and the declaration of phase 6 in the city, which prevented the team from entering the hospital to collect data.

Data collection was performed by a team of six nurses, previously trained in order to ensure the same standard for conducting the interviews. Eligible women were daily identified by the collector through the admission map, and those to be invited to participate were selected with the app Sorteador®. Data collection took place in two steps: 1^st^ – data characterizing the participants (socio-demographic, obstetric, neonatal, and COVID-19) collected directly from institutional records and 2^nd^ – interview conducted in the private patient room. Triggering question: "How was it for you to experience pregnancy during the COVID-19 pandemic?". The interviews, with an average duration of fifteen to thirty minutes, were recorded and transcribed in full and stored at the Research Electronic Data Capture (RedCap^®^) of the research, which only study participants could access, using a unique password.

Klaus Krippendorff's Content Analysis,^(11)^ the theoretical-methodological framework adopted in the study, is a scientific method with a complex, hierarchical structure, which systematically reviews the content of the interviews, aiming at understanding the meaning of their symbolism, considering the context where they were produced. The analysis is started with a deep dive in the data with deep reading of the interviews, observing the smallest unit of meaning, referred to as unitizing by the author, until a broader theme is obtained, from specific to general, grouping categories by similarity and meaning, until the conclusion on the content's significance is found. The construction of the final report must include tree diagrams called dendrograms, which identify the line of reasoning followed by the authors regarding the emerging themes. Although the theoretical saturation of the data occurred prior to the analysis of the 106 interviews, the authors chose to review the entire material, due to the peculiarity of the population and the pandemic setting. The data analysis was performed by two analysts and, in case of discrepancies, a third analyst participated in the classification consensus. The Consolidated Criteria for Reporting Qualitative Research (COREQ) checklist was used in the design of this study.^(11-13)^

This study is part of a larger study, entitled Perinatal Mental Health in the Covid-19 Pandemic, and was conducted in accordance with the Declaration of Helsinki and approved by the Research Ethics Committees of the Federal University of São Paulo, opinion no. 4,271,336 - CAAE 36783720.0.0000.9887, and of the co-participating institution São Luiz Hospital and Maternity, opinion no. 4,650,135 - CAAE 36783720.0.3001.0087. All participants signed two copies of the Informed Consent Form (ICF), one being kept by the research team and the other given to the postpartum woman.

## Results

One hundred and thirty-six women were eligible for this study, 118 agreed to participate, but 12 interviewees were excluded due to issues with the recording (poor audibility or corrupted file). The sample was set at 106 participants with an average age of 34 years and a higher education degree, who lived with their partners and were formal workers, with an annual income of approximately U$16,800.00 at the time. Although the majority of them considered that COVID-19 interfered with their pregnancy, they also considered that the pregnancy occurred at the right time. The majority of them were multiparous women, with usual pregnancy risk. Around 59% had a cesarean section, and their companions during birth and hospitalization were their partners. The average gestational age was around 38 weeks. The babies, mostly boys, weighed an average of 3,200 grams and almost 80% were exclusively breastfed during the period of the interview. All variables are represented on table 1.

**Table 1:** Characterization of the study population, according to socioeconomic, obstetric and birth data

Characteristic		n=106 (%)
Age (years) Mean ±SD		34.075±4.43
Education Level		
	College degree	92.45
	High school	7.55
Marital Status		
	Has a partner and lives with him/her	87.73
	Has a partner and does not live with him/her	0.94
	No partner	1.88
	Uninformed	9.43
Current employment status		
	Formal	77.35
	Informal	6.60
	Housewife	5.66
	Uninformed	10.37
Family income* (monthly)		
	01 ┤ 03	1.9
	03 ┤ 05	12.3
	05 ┤ 07	14.2
	≥ 07	71.7
Interference of COVID-19 on pregnancy		
	No interference	33.96
	Major interference	25.47
	Little interference	31.13
	Uninformed	9.43
Timing of pregnancy		
	Right moment	76.42
	Exactly at the right time	11.32
	Wrong timing	2.83
Obstetric history		
	Average pregnancies ±SD	1.754±1.005
	Average births ±SD	1.358±0.632
Abortions		
	Yes	23.58
	No	73.58
	Uninformed	2.83

* Current minimum wage, at the average Dollar exchange rate at the time $250.00

The analysis of the interviews revealed, through four themes, how the subjects experienced pregnancy during the pandemic. The themes were: Fear, taking care and celebrating pregnancy: adjusting to the new reality, Harms of Isolation, and Benefits of Isolation.

### Theme 1: Fear

*It was very hard, because I was very worried about catching COVID, so I actually had to change my entire routine and stay trapped at the house, always anxious about any symptom I had […] already thinking I had COVID. Thank God I didn't have any positive results, […] but I think it impacted my entire pregnancy 100%. (E131)*

The majority of the women investigated in this study reported that their pregnancy occurred at the right time in their lives. However, the discovery of the pregnancy was received with fear and concern given the pandemic scenario and the uncertainty regarding the continuity of prenatal care, and the risks and impact of COVID-19 on maternal health and/or fetal/neonatal development, which caused a feeling of dread in some couples.

Pregnant women were not initially considered a risk group; however, even before they were, the fear that contamination could impact the baby's development was already a reality for women. The appearance and publication in the media of the first cases of infected pregnant women and the variety of signs and symptoms presented, as well as the lack of information about possible complications for mother and fetus/child, were very difficult for those investigated, increasing the feeling of insecurity in the face of pregnancy.

Scared, women doubled their caution and adopted strategies and changes in their routine to avoid contamination. They restricted visits, leaving home, interaction with other people, canceled vacations and isolated themselves as much as possible, with the intention of protecting themselves and their babies as much as possible.

In cases where a breach in isolation was imperative, whether due to the obligation to work or the need to keep prenatal appointments and tests, the expecting mothers reported experiencing feelings of heightened tension, anxiety, and stress. The fear of bringing the disease home was very present in the reports of working mothers, particularly healthcare professionals.

Fear surrounded the pregnancy and the lack of perspective regarding the duration of the pandemic was an important stressor in the emotional state of these women. The fear of having a complication during childbirth/postpartum, of not being able to be with the baby after birth and/or not being able to breastfeed left them anxious and distressed, in addition to producing a "certain tension", translated by many as a state of permanent irritability. Pregnancy is inherently a period of fear and uncertainty for women, such as fear of miscarriage, malformation, and childbirth. But the pandemic heightened all these fears due to the constant feeling of living at risk.

For multiparous women, comparisons with previous pregnancies came to the fore, placing the current pregnancy as more challenging. As for first-time pregnant women, the feeling mentioned was that the situation was maddening, as they had to simultaneously deal with the symptoms and changes of a pregnancy and the pandemic.

Finally, the pregnant women indicated a fear that they were forced to deal with daily: the fear of death. Whether it was the death of relatives, the baby or their own death, expecting mothers had to deal with the constant reminder that thousands of people died every day and these numbers were ostensibly televised. In spite of the feeling of security for being in a private hospital with all necessary infrastructure, equipment, and personnel, the fear of the healthcare system collapsing and the potential unavailability of care, if necessary, was a reality for many of them.

**Dendrogram 1:**
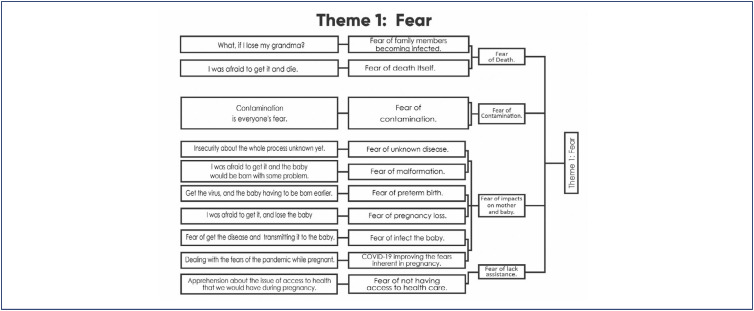
Fear

### Theme 2. Taking care and celebrating pregnancy: adjusting to the new reality

*We had to adjust and, in the end, everything worked out, but it certainly would have been very different if it weren't for the pandemic. We would have had a party for the family, a baby shower, you know, those things you normally do… none of that happened. (I115)*

Being pregnant during the pandemic was a different experience than most women imagined for this moment. Women expected a celebrated pregnancy, shared with their family and loved ones. They hoped to have parties, show the changes in their belly, and for people to see them pregnant. However, the social isolation imposed as a strategy to protect against contagion required distancing from close friends and, especially, family members.

Pregnant women had to adapt to the new reality, a solitary pregnancy shared only through virtual contact and without support from family members. Worried, insecure, and afraid of being contaminated and, consequently, of compromising their health or their baby's health, women sought strategies that could be controlled and changes in their daily routine inside and outside the home.

The only situations of disrupted isolation were related to keeping prenatal appointments and going to the supermarket, the latter specifically delegated to the partner. The need to ensure that the pregnancy was healthy overcame the fear of leaving home, and in these trips, caring, exposure, and fear were mixed. Leaving home was done with great caution, wearing masks, and with frequent sanitization of hands and surfaces, as a solution to alleviate the concerns and stress experienced in these moments.

Women tried to maintain confinement for as long as possible, experiencing pregnancy in a lonely way, and there were many reports of frustration. The pregnancy went by and nobody saw the belly. Technology allowed the maintenance of contact and the exchange of experiences between the pregnant woman and her family and/or friends, while ensuring the safety of isolation. However, this wasn't the type of contact they craved. Rituals commonly carried out during pregnancy, such as baby showers, were adapted to a new remote model, via video conferences, so that the family could take part in the festivities. The trousseau was also entirely bought online.

The frustration of sharing pregnancy, childbirth, and other moments during this phase, virtually, was intense for these women.

**Dendrogram 2:**
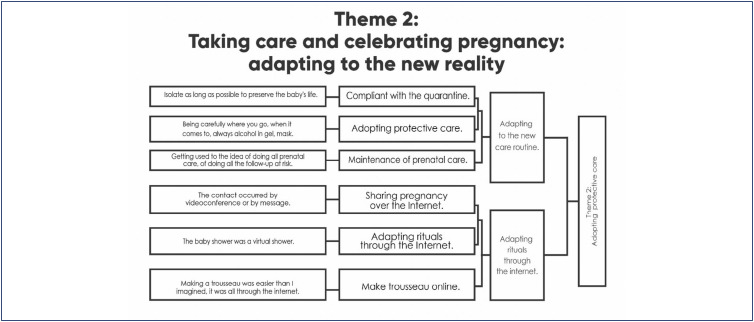
Taking care and celebrating pregnancy: adapting to the new reality

### Theme 3. Harms of Isolation

*[…] we wait so long for a child to arrive, we plan… And then suddenly we are alone, it feels like it was not celebrated as expected. I think this really got me, because at the same time you’re expecting a child so much and not celebrating the arrival of that child with your family, with everyone around, was very difficult. So, I was very discouraged, very unmotivated to do these things. (I113)*

Pregnancy and the arrival of a child are important events in the lives of women, their partners, and their families. Waiting for the best moment to become pregnant and planning every detail of this experience was a dream for many women. In this sense, the isolation imposed during the pandemic brought frustration and, for some, the lack of interaction brought the feeling that this child was not celebrated, making it seem like their child was not welcomed by family members at the time and in the way that they deserved.

The lack of sharing gave some women the feeling that they were not pregnant, or even that it had passed quickly, in the blink of an eye, just at the moment when they felt most fulfilled in their lives.

The pandemic took away from them the "glamor of pregnancy", the joy of the celebrations, the welcome and love emanated by their family during this period when they had so many expectations. It was very difficult to accept the reality experienced without this social validation.

Pregnancy is a phenomenon experienced as a community, and usually shared with joy by those closest to you. Suddenly, all contact stopped, the entire support system was suppressed, and the expecting mother saw herself alone and, sometimes, desperate with the physical and emotional changes that characterize this period. The presence that was most missed, for some women, was the parents, but especially their own mothers. The partner was the person who was closest to the pregnant woman throughout the isolation and, although they did not replace the maternal presence, they could be perceived as an important support figure.

If, on the one hand, being confined at home alleviated the fear of contamination, on the other hand, it increased the women's workload. The demands of the different social roles played by women with a total fusion of work time and free time, without delimitation between one thing and another, ended up causing physical and mental exhaustion, leading these women to develop pathologies such as anxiety, depression, insomnia, burnout, high blood pressure, and pre-eclampsia. In this scenario, some pregnant women sought specialized care, starting or resuming psychotherapy and drug treatment to deal with the difficulties of this period.

**Dendrogram 3:**
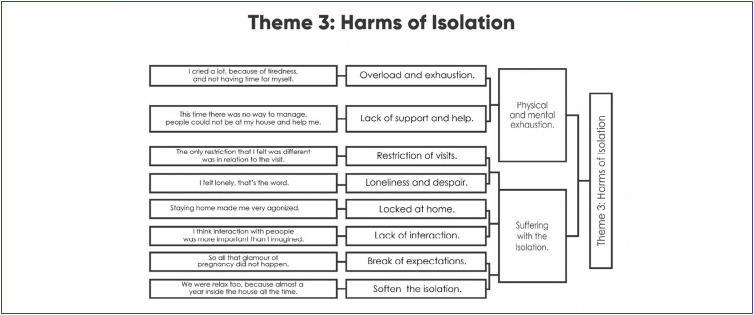
Harms of Isolation

### Theme 4. Benefits of Isolation

*[…] the isolation itself in my specific case wasn't bad, in my case it wasn't bad, because I was able to give myself time to actually be pregnant, peace of mind inside my home. (I02)*

Despite being a drastic measure, social isolation proved to be a period of great opportunities and benefits for some pregnant women who, despite being aware of the difficulties of the period, full of losses and tragedies, were able to live beneficial experiences that would not have been possible if they had not been in the pandemic period.

When working remotely, these women decided that this would be the ideal time to become pregnant, considering themselves blessed for generating life amidst so many deaths. By becoming pregnant in isolation, the women were able to feel all the effects and complications inherent to pregnancy in the comfort of their home, freeing themselves from public transportation and the long commutes that they would normally be forced to make on a daily basis. This was reported as a great relief.

Being home during the pregnancy allowed women to turn their full attention to the pregnancy, dedicating themselves to the soon-to-be-born child. Their pregnancy was enjoyed, as there was plenty of time for self-care. The relationship with the partner was improved, with the couple reconnecting and improving their partnership, with a mutual dedication to the pregnancy.

The calm, peaceful and relaxing environment provided by the home proved to be very beneficial to the women, with some of them directly pointing to a fulfilling isolation period as the reason for having a successful pregnancy. This feeling of peacefulness was so intense that one of the interviewees described pregnancy in isolation as a nine-month vacation. Something relevant for a portion of the interviewees was avoiding contact with unwanted people. Taking advantage of social isolation, the women selected the people they wanted to spend time with during the development of their pregnancy.

**Dendrogram 4:**
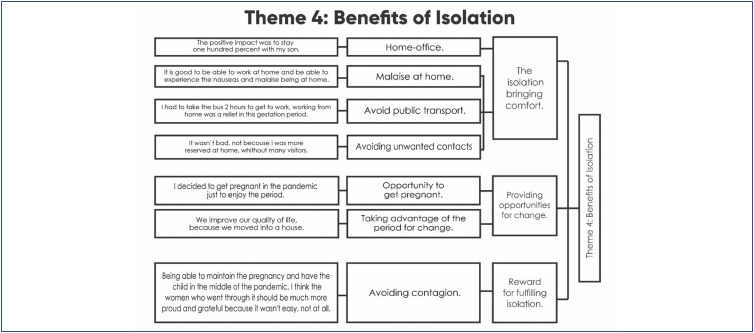
Benefits of Isolation

## Discussion

The COVID-19 pandemic has led to countless changes in people's daily lives. Aiming at preventing people from being infected by the new coronavirus, social distancing caused a significant change in several aspects of this pregnant woman's life, affecting her way of working, her relationships with people, and her way of experiencing pregnancy.

The first feeling associated to the pandemic was fear. Fear took over these women. The lack of knowledge about the disease and the potential impacts it could have on the baby caused real fear in them, particularly since Brazil was the country with the highest number of pregnant women who died from COVID-19 in the world.^(9)^

Fear in the pandemic had several versions. Fear for herself, fear for the baby, and fear for loved ones were the most prevalent. This finding is in line with several international studies that show that fear of the unknown and its potential risks to maternal and fetal health were described by women around the world.^(14-26)^

The fear that the health system would simply become saturated and there would be no more hospital beds, whether in the private or public system, was another fact that affected women in the study. This fear made pregnant women question their place in the pandemic, sometimes including themselves as a risk group, sometimes considering themselves privileged, as they were in a high complexity hospital in the supplemental health system, unlike thousands of public system-dependent pregnant women who died due to lack of assistance and/or equipment. The answer to this question is supported by the statistical analysis of national maternal mortality, which shows that women with less education and at extreme ages also have higher maternal mortality.^(10)^

Considering this data and crossing them with the characteristics of the population of this study, comprising mainly women with higher education and a mean age of 34 years, we can assess that these women actually had greater protection as they were not part of the "risk group" of maternal mortality in Brazil.^(10)^

Along with the fear of the new disease came the need to act to slow the spread of the coronavirus. As it was something completely new, without scientifically proven medication or treatment and transmitted through contact with contaminated people and surfaces, the feeling was that, in addition to being invisible, "the enemy" was everywhere. Faced with all this uncertainty, such as the form of infection or what the outcome would be if they were infected, women chose to adopt the recommended care and adapt it to their routine.^(15-17,21,22)^

Pregnancy, previously a community phenomenon, had to be faced alone, and the lack of contact with relatives and, felt in a more overwhelming manner, the lack of the pregnant woman's mother, was a great suffering for these women, marking pregnancy in a negative way during the pandemic. Being accompanied by the maternal grandmother during postpartum is a common custom expected by women, and this lack was also felt by women around the world.^(23,24)^

Vaccination, considered the main event for containment of the widespread transmission of the virus, only began in January, 2021, for specific priority groups, and, for pregnant women, vaccination only started in May of the same year, but still only included those who had a comorbidity. Therefore, during most of the data collection period, the women lived with the expectation of the discovery of the vaccine and its mass application.^(27,28)^

The lack of delimitation between work, household tasks and routine plus the increase in activities and the lack of escape, such as walks and physical exercise, caused mental disorders to surface, with pregnant women being diagnosed with anxiety, depression, burnout syndrome, and insomnia with a range of symptoms, from mild sadness to a complete lack of control of the body and thoughts, requiring the prescription of psychotropic drugs to treat this overworked mother.^(14-18,26)^

Pregnancy experienced in the privacy of the home, with only virtual sharing, became an abstract phenomenon in the outside world and women were deprived of the pregnancy planned by them. Their dream pregnancy was stolen by the pandemic.^(16-25)^

This breach of expectations of dream pregnancy versus pregnancy during pandemic made some women feel that this unique moment was not experienced according to the standard recognized by them of social sharing and, therefore, for some, this deviation from festive rituals and the fact that family and friends did not see the evolution of the mother's belly caused a feeling of invalidation, with strong reports that the fact of not sharing the pregnancy caused the feeling that it had not existed. This lack of "completeness" in the pregnancy experience, including when compared to the pregnancies of older children, was reported almost as a confession of guilt for having done "less" for the child, as if the limitation of celebrations implied less love and care for the child born now.

Two Brazilian studies found similar results to the present study. A study conducted in the São Paulo state inland area, only with infected pregnant women, found similar results, worsened by the experience of diagnosis and hospitalization. The women in this study also felt fear of premature birth, fear for their babies, sadness due to isolation, and fear of dying from the disease, but there was another specific feeling: guilt for having been infected. The other study, conducted in different locations in different states, also found the same perceptions; however, due to the fact that they surveyed a population using the public health system, a great concern was detected regarding family income, which was impacted by the pandemic.^(25,26)^

Regardless of the different nuances depending on the populations studied, the perceptions of fear of infection, uncertainty about the disease, and sadness caused by isolation seem to be universal among pregnant women in Brazil and around the world.^(25,26)^

Although they were afraid, women notably prioritized ensuring the baby's well-being and development over their fear, and going to prenatal appointments was mentioned as the main reason for leaving complete isolation. Despite all the fear about going to health services (clinics, hospitals, laboratories), leaving a completely safe and protected environment to be in the same environment where the virus could potentially also be present, these women faced the fear in favor of obtaining care for their pregnancy.

However, isolation also brought positive experiences, increasing the calmness of these women, which is "the good side of the pandemic". It was evident, for instance, that the type of remote work applied by companies brought a range of benefits to these women, bringing comfort that they would not have in regular times of in-person work. Another reported fact was the selection of people to live with during pregnancy, avoiding unwanted contacts. This fact has also been reported in other international studies.^(16,18,19,23)^

The possibility of taking rest breaks, dedicating time to preparing for the pregnancy with reading, shopping for the baby, or other pleasurable activities, in addition to being closer to the partner, was highlighted. Together, these advantages led to the experience of a more peaceful and comfortable pregnancy, in complete contrast to what would be "normal" in their daily lives.^(16,23)^

The confinement period served to build and solidify the role of mother and father, as it gave the couple the opportunity to fully dedicate themselves to the pregnancy. The presence of the husband at home favored his participation during pregnancy, strengthening the couple's bond.^(16,22)^

In addition to being a historical document about the experience of pregnancy during the pandemic, the study brings important contributions for the understanding around the Perinatal Mental Health scenario and its post-pandemic legacy, seeing as the findings align with studies worldwide, emphasizing their significance.

The study could not generalize the experience of the investigated population to other pregnant women, considering the population size and the significant social disparity in Brazil.

## Conclusion

The findings of this study are in line with what has been reported in international studies with a similar topic. Fear, the solitary experience without social support during pregnancy, adjustments to the new prenatal routine, and the ritual of pregnancy were events and feelings that were part of pregnancy during the pandemic globally. And experiencing pregnancy this way, so far from what was recognized as "being pregnant", was lived with a feeling of regret and mourning, as pregnancy is a unique moment in a woman's life, and the pandemic took away the opportunity to feel and live their dream pregnancy. However, the pandemic also benefited this woman, who despite the distance from loved ones, was able to live with more peace and relaxation, without having to experience the routines of commuting and daily schedules, allowing for a greater bond with her partner and greater dedication to the new baby and family. In regular times, such benefits would not exist or would not be experienced to the same extent.
